# Predictors of English Health Literacy among U.S. Hispanic Immigrants: The importance of language, bilingualism and sociolinguistic environment

**DOI:** 10.5130/lns.v24i1.4900

**Published:** 2016

**Authors:** Holly E. Jacobson, Lauren Hund, Francisco Soto Mas

**Affiliations:** 1 Department of Linguistics, MSC 03 2130, 1 University of New Mexico, Albuquerque, NM 87131-0001, Phone: (505) 277-6353 (Voice), Fax: (505) 277-6355, jacobson@unm.edu; 2 Department of Family and Community Medicine, Public Health Program, University of New Mexico, lhund@salud.unm.edu; fsotomas@salud.unm.edu

## Abstract

In the United States, data confirm that Spanish-speaking immigrants are particularly affected by the negative health outcomes associated with low health literacy. Although the literature points to variables such as age, educational background and language, only a few studies have investigated the factors that may influence health literacy in this group. Similarly, the role that bilingualism and/or multilingualism play in health literacy assessment continues to be an issue in need of further research. The purpose of this study was to examine the predictors of English health literacy among adult Hispanic immigrants whose self-reported primary language is Spanish, but who live and function in a bilingual community. It also explored issues related to the language of the instrument. An analysis of data collected through a randomized controlled study was conducted. Results identified English proficiency as the strongest predictor of health literacy (p < 0.001). The results further point to the importance of primary and secondary language in the assessment of heath literacy level. This study raises many questions in need of further investigation to clarify how language proficiency and sociolinguistic environment affect health literacy in language minority adults; proposes language approaches that may be more appropriate for measuring health literacy in these populations; and recommends further place-based research to determine whether the connection between language proficiency and health is generalizable to border communities.

## Background

The 2003 National Assessment of Adult Literacy (NAAL) found that Hispanics in the United States (U.S.) had lower levels of health literacy compared to other population groups (U. S. Department of Education 2006). However, recent reports and research studies have concluded that there is a need for more recent and reliable data on health literacy among certain groups, including Hispanics and Spanish-speaking adults (Berkman, et al 2011, Koskan, Friedman & Hilfinger Messias 2010, Soto Mas, et al 2015, Soto Mas, Mein, Fuentes, Thatcher & Balcázar 2013, Soto Mas, Ji, Fuentes & Tinajero 2015, U.S. Department of Health and Human Services 2010). The national-level data is more than 10 years old, and there is a scarcity of current information on the health literacy levels of populations with limited English language skills (U.S. Department of Health and Human Services 2010). Although the 2003 NAAL embedded most of the health-related questions into the main section of the questionnaire, it may not be an appropriate tool for assessing health literacy among non-English speakers. First, NAAL measures English oral fluency and “how well Americans perform tasks with printed materials similar to those they encounter in their daily lives at work, at home, and in the community,” which may include balancing a checkbook (quantitative literacy), filling out a job application (document literacy), or finding information in a news article (prose literacy) (National Center for Education Statistics [NCES] n.d.). Under this framework, English proficiency, or the lack thereof, becomes a confounding factor in the assessment of health literacy. Similarly, people who are not originally from the U.S. may find scenarios and tasks portrayed by NAAL foreign to them, which adds an additional threat to the internal validity of the instrument.

Aside from the data generated by the 2003 NAAL, only regional U.S. studies have assessed the health literacy level of Hispanics, mostly in clinical settings, and with conflicting results. Studies in North Carolina, New York, and California found high prevalence of low health literacy among male and female patients (Brice, et al 2008, Garbers, Schmitt, Rappa & Chiasson 2010, Sudore, et al 2009). To the contrary, a study with primary care patients on the U.S.-Mexico border found that more than 98% had adequate health literacy (Penaranda, Diaz, Noriega & Shokar 2012). A more recent study with Hispanic college students on the U.S.-Mexico border also found higher levels of health literacy in this group than in the general Hispanic population, and similar to educated U.S. adults (Mas, Jacobson& Dong 2014).

There are also inconsistencies across studies in terms of the factors that have been identified as possibly influencing health literacy among Hispanics. In the general U.S. population, national data identified gender, age, educational level, and language as relevant variables affecting health literacy level. The 2003 NAAL found that women had higher average health literacy than men; adults 65 years-of-age and older had lower health literacy compared to younger adults; and average health literacy increased with higher level of educational attainment (Kutner, Greenberg, Jin& Paulsen 2006). In the case of Hispanics, however, there are conflicting results due in part to the fact that these variables have only been explored in small studies with different demographic groups. One study found that education was a significant predictor of health literacy but that age, gender, income, and citizenship status were not (Boyas 2013). Two recent studies suggested that education and age may have an effect on health literacy (Mas, Jacobson & Dong 2014, Soto Mas, Ji, Fuentes & Tinajero 2015). Although these discrepancies may be related to the disparate designs, sub-populations and instruments generally used in studies with Hispanics (Koskan, Friedman & Hilfinger Messias 2010, Soto Mas, et al 2015), there are also relevant conceptual issues relating to health literacy that must be further explored. For instance, health literacy has no single, standard definition. At times it is defined as simply the ability to read and write, and in other cases it is more comprehensively linked to socio-cultural and political change (Soto Mas, Jacobson, Balcázar 2015). Methodological approaches vary according to the definition used by the study. Similarly, the roles that both primary (L1) and secondary (L2) language use and proficiency play in health literacy assessment in bilingual communities has not been researched or discussed in the literature. In the U.S., this is particularly relevant for Spanish speakers, as more than 37 million people speak Spanish, and approximately half of them speak English “less than very well”, with the rest speaking English at varying levels of proficiency (Ryan 2013). In addition, no research to date has explored the interaction within bilinguals of L1 and L2 languages, and impact on health literacy levels. Future research should explore the role L1 literacy plays as a predictor variable of L2 health literacy.

Another issue to consider is the fact that health literacy has generally been defined as the ability to understand *English* health information (Kirsch, Jungeblut, Jenkins & Kolstad 1993, Kutner, Greenberg, Jin& Paulsen 2006, Nielsen-Bohlman, Panzer & Kindig 2004), and is a construct that is embedded within the U.S. healthcare system, which is predominately English-speaking. Studies have found that even when health literacy assessments are conducted in Spanish, participants whose L1 is self-reported as Spanish have lower health literacy than L1 English speakers (Brice, et al 2008, Garbers, Schmitt, Rappa & Chiasson 2010, Sentell & Braun 2012, Williams, Parker & Baker 1995). Researchers have debated whether or not it is appropriate to measure health literacy in other than the L1 of the participant, and recommend that studies with Hispanics control for language use (that is, collect data on the languages used by participants for different purposes and contexts) and language proficiency (Koskan, Friedman& Hilfinger Messias 2010, Mas, Jacobson & Dong 2014, Soto Mas, et al 2015, Soto Mas, Ji, Fuentes & Tinajero 2015). In fact, NAAL includes a questionnaire with items on language background (NCES n.d.). However, these variables are difficult to control without going beyond self-report on language use and conducting proficiency testing in the participants' languages. And even if these variables were controlled, the question still remains as to how the interaction between two languages in a bilingual individual impacts the results, or, just as importantly, the interpretation of the results, of commonly used health literacy assessments, such as the Test of Functional Health Literacy in Adults (TOFHLA) and the Program for the International Assessment of Adult Competencies (PIAAC). TOFHLA measures functional health literacy by assessing numeracy and reading comprehension using actual health-related materials such as prescription bottle labels and appointment slips, and is available in both English and Spanish (Peppercorn Books & Press, Inc.). PIAAC assesses literacy by measuring “understanding, evaluating, using, and engaging with written texts to participate in society”. Although PIAAC is part of an international program involving more than 20 countries and is available in multiple languages, it is administered in the “official language” of each of the participating countries (Organisation for Economic Co-operation and Development [OECD] 2012).

In the Hispanic communities that exist throughout the U.S. where the majority of Spanish speakers are bilingual to some degree along a continuum, it is not clear which health literacy assessment tool should be used. Hispanics in the U.S. cannot be reduced to such simple categories as "Limited English Proficient" as is currently the practice: language use among Hispanic groups throughout the U.S. is complex and dynamic, and requires much deeper exploration in order to better understand health literacy among these groups. It often is not clear which literacy tests, the English test or the Spanish test, will provide the most reliable health literacy score in bilinguals. For example, which test will provide the most reliable score for a bilingual who speaks Spanish primarily at home, but uses English at work, or for the bilingual who obtained a high school equivalent education in a Spanish-speaking country, but has resided in the U.S. for 10 years? There is great complexity involved in measuring health literacy among bilinguals, leading to other unexplored questions: does having low health literacy in Spanish constitute low health literacy in English and vice versa? Does the score on a test of health literacy in Spanish predict how a person will access or process information in English in an English-speaking context? In other words, if a person has high health literacy in Spanish, what does this mean when faced with the challenges of accessing information in an English-speaking context? These and a myriad others are questions that have not been addressed in the literature.

It is such complexity that justified this study. The purpose was to examine the predictors of health literacy, assessed in English, among adult Hispanic immigrants. Variables of age, sex, years living in the U.S., education level, whether participants attended English classes and school or college in the U.S., and language history and preferences were included in the analysis. The study involved immigrants whose L1 is Spanish but who show varying degrees of proficiency in English. In other words, these immigrants fall along a bilingual continuum, according to the demographic information provided. Issues related to the language of the instrument, specifically the significance of the language of the test and the sociolinguistic environment of the community in which the data were collected are also explored.

This was part of a larger study conducted in a southern city on the U.S.-Mexico border that assessed the effectiveness of a curriculum in improving health literacy and English proficiency among Hispanic adults. Information on the curriculum and the results of the intervention have been reported previously (Soto Mas, Mein, Fuentes, Thatcher & Balcázar 2013, Soto Mas, Ji, Fuentes & Tinajero 2015). The study obtained approval by the Institutional Review Board and all participants signed an informed consent.

## Methods

This study involved an analysis of baseline data collected for a randomized controlled study and included only self-reported immigrants.

### Participants

Participants were recruited from the community through local Spanish media. The original inclusion criteria for the larger study included adults (21+ years of age) with Spanish as their L1, able to read and write Spanish, and self-reported as having no previous participation in a formal health/cardiovascular education/prevention program. Since the interest of the study was to assess health literacy in English, only people with a low to intermediate level of English proficiency -able to read, write and speak English at a basic level- were included in the study. For this analysis, the number of eligible cases was adjusted to include only those who were born outside the U.S. as a means of controlling for immigration status.

### Data Collection and Measures

Prior to inclusion, all individuals who met the criteria and indicated interest in participating were screened for English proficiency. The Combined English Language Skills Assessment (CELSA) (Association of Classroom Teacher Testers, CA) was used. CELSA is a standard computerized proficiency test that measures grammatical ability and understanding of meaning in a typical reading context, and is generally used as a placement test in foreign language programs. The test is written in English and all instructions are given in English only. It establishes three levels of language proficiency based on the obtained score: 90–102 level 1, 103–107 level 2, and 108–114 level 3 (Thompson 1994).

Health literacy was assessed using the English version of the TOFHLA, which has proven to be a valid and reliable instrument for testing literacy in a particular domain (healthcare) in which particular domain-specific documents are used, including, for example pharmaceutical labels and patient education materials (Parker, Baker, Williams & Nurss 1995). Assessment of health-related reading fluency is essential because, as a predictor variable, it is more powerful than a measure of general reading fluency in “detecting associations with health outcomes” (Baker, 2006). In addition, it is not possible to simply assume a correlation between general reading fluency and health literacy. TOFHLA classifies participants in three categories according to their total score: inadequate functional health literacy, marginal functional health literacy, and adequate functional health literacy.

Participants were also asked to complete a brief bilingual demographic questionnaire that included questions on: age, sex, years living in the U.S., education level, whether they attended English classes and school or college in the U.S., and language history and preferences (see Table 1 below).

### Data Analysis

Data quality included crosschecking 100% of the cases. Missing data was minimal. For this study, all analyses were conducted in Stata v13 (StataCorp, 2013) and using complete cases only. The original study included 181 participants. This analysis was conducted with 144 participants after excluding incomplete cases and those who were born in the U.S. and did not meet the immigrant criterion (10 cases).

Associations between demographic characteristics, English proficiency, and health literacy were quantified. The continuous outcome measures were total TOFHLA scores (range 0–100), weighted numeracy scores (range 0–50), and reading comprehension scores (range 0–50). Total TOFHLA scores were categorized into inadequate functional health literacy (score 0–59), marginal functional health literacy (60–74), and adequate functional health literacy (75–100).

Demographic predictors of interest in the study included all demographic variables and English proficiency (CELSA score) at baseline. Simple linear regression models were used to calculate differences in means across levels of the predictors. For each continuous outcome, changes were estimated as a function of each predictor, along with confidence intervals. For continuous predictors, the linear model was compared to models with higher-order polynomial terms using an F-test. For ordinal categorical variables, the saturated model was compared to a linear trend model using an F-test. Regression coefficient F-tests were used to test for differences in the average outcome as a function of each predictor in the univariate analysis. In a multivariate analysis, a multiple linear regression model was used to summarize effects of English proficiency, controlling for the other predictors. Non-significant terms were dropped from the multiple regression model using a backwards stepwise procedure with a p-value threshold of 0.1.

Differences were examined in the TOFHLA categories across quartiles of English proficiency scores and testing for an association between the categorical outcome and quartiles using a Pearson chi-square test. All hypothesis tests were conducted at the 0.05 level of significance and confidence intervals at the 95% level.

It is important to note that although the original study implemented a randomized control, pretest and posttest design, this analysis was conducted only on the baseline data. The aim was to make a prediction, rather than establishing a causal inference.

## Results

Demographic characteristics of the sample are included in Table 1. The majority were 31 to 60 years of age (85%), female (78%), and had been living in the U.S. for eight or more years (74%). More than 80% graduated from high school or had a higher degree, and less than 40% attended school/college in the U.S. Regarding language background and preferences, a high majority spoke Spanish at home (81%). Descriptive statistics for test scores are included in Table 2.

TOFHLA results yielded an overall marginal functional health literacy level (mean score 63.7), with 51 (35.4%) participants at the inadequate level; 52 (36.1%) at the marginal level, and 41 (28.5%) at the adequate level. Reading comprehension mean score was higher (33.8) than that for numeracy (29.9).

There was no evidence of non-linearities in the relationship between the continuous variables (English language proficiency and age) and any of the outcomes. Therefore, the continuous variables were included as linear terms in the regression models. The estimated mean test scores, with 95% confidence intervals, as a function of the predictor variables are shown in Table 3. English proficiency was the strongest predictor of health literacy scores. The estimated average TOFHLA score increased linearly with English proficiency score (p < 0.001). As shown, 1 standard deviation increase in English proficiency resulted in an estimated 10 point increase in TOFHLA score. There was no evidence of a difference in mean TOFHLA score and any of the other variables. Numeracy and reading comprehension scores also increased with English proficiency score. Average reading comprehension score was higher in those with more than 1 year of English language courses. There was no evidence of differences in mean reading or numeracy scores across levels of any of the other variables (though age was almost a statistically significant predictor of numeracy).

Results from the multiple linear regression models are in Table 4. After implementing the stepwise selection procedure, age and English proficiency were the only variables retained in the linear regression models for total TOFHLA score and numeracy score; and English proficiency was the only variable retained in the model for the reading comprehension score. The relationship between English proficiency and the three continuous outcomes, total TOFHLA, weighted numeracy, and reading comprehension, remained statistically significant (p < 0.001). After adjusting for English proficiency score, the magnitudes of the age-total TOFHLA and age-numeracy score associations increase; specifically, the average numeracy score decreases on average with age (p = 0.02), resulting in a trend of a decrease in total TOFHLA scores with age (p = 0.07). There was no evidence of a difference in reading comprehension score as a function of age.

TOFHLA category frequencies as a function of quartiles of English language score are shown in Table 5. The same pattern of monotonic increase in TOFHLA performance as a function of English language score is evident.

## Discussion

To our knowledge, this is one of few studies that have explored predictors of English health literacy specifically among U.S. adult Hispanic immigrant bilinguals. There is consensus among experts on the need for further research involving language minorities and exploring the relationship between health literacy and limited English proficiency (McKee & Paasche-Orlow 2012, Sentell & Braun 2012). Consistent with the literature, the study emphasizes language history and preferences. The demographic data suggest a continuum of bilingualism in this sample, as influenced by number of years living in the U.S.; experience with the U.S. education system; and amount of time in English classes. A strength of the study was the fact that English proficiency was assessed through a standard test, rather than self-reported. In addition, by including only participants born outside of the U.S. it was assumed that participants had a high proficiency in oral Spanish, although future studies would benefit from also administering a test in Spanish, as suggested by the varying levels of education in this sample. Additionally, the study individually explored two key health literacy domains: reading comprehension and numeracy. Finally, analyses included absolute TOFHLA score as well as categorical results or health literacy level. It might appear that using an English instrument would compromise the validity of the test when administered to Spanish speakers, as is argued in the literature. However, it is essential to consider that the use of a Spanish instrument would be equally problematic, unless all of the participants being assessed have no fluency in English (such as being recent arrivals to the U.S.).

Overall, TOFHLA results indicated marginal functional health literacy in this sample, when measured in English. This result is consistent with existing national data. The 2003 NAAL found that 66% of Hispanics had “basic” or “below basic” health literacy (Kutner, Greenberg, Jin& Paulsen 2006), and that 13.8% of Medicare managed-care Spanish speaking enrollees had inadequate health literacy (Wolf, Gazmararian & Baker 2007). However, it is important to remember that small regional and clinical studies with Hispanics have provided inconsistent results, some finding a high prevalence of limited health literacy (Brice, et al 2008, Garbers, Schmitt, Rappa & Chiasson 2010, Sudore, et al 2009), and others finding a high percentage of people with adequate health literacy (Mas, Jacobson & Dong 2014, Penaranda, Diaz, Noriega & Shokar 2012,). As mentioned previously, these discrepancies may be related to a number of theoretical and methodological issues that must be further explored, including geographical and sociolinguistic factors.

This study identified English proficiency as the strongest predictor of TOFHLA scores among participants, including numeracy and reading comprehension scores. Results indicate that time attending English language courses may also constitute a relevant factor affecting functional English health literacy. Categorical results confirmed the positive effect of English proficiency on health literacy (Table 5). These results are consistent with previous literature emphasizing the importance of English language proficiency in health literacy (McKee & Paasche-Orlow 2012, Pippins, Alegría & Haas 2007, Sentell & Braun 2012). This study also found evidence that younger participants obtained higher numeracy scores (after adjusting for English proficiency). Thus, age may be a relevant demographic factor associated with functional health literacy and numeracy in this population. A previous study with Hispanics in Arkansas found that age and gender were not significant predictors of health literacy scores (Boyas 2013), however it is not clear whether that study controlled for immigrant status or proficiency in English and Spanish.

As would be expected, completing the test in a language in which the participants were not highly proficient seems to have negatively affected the outcome of the test. The typical argument to recommend testing health literacy in English is that English is the dominant language in the healthcare system in the U.S., and that there is therefore a need for the general public to understand English health information (Kirsch, Jungeblut, Jenkins & Kolstad 1993, Kutner, Greenberg, Jin& Paulsen 2006, Nielsen-Bohlman, Panzer & Kindig 2004). In addition to the obvious question of whether similar results would have been obtained if the Spanish TOHFLA had been implemented, it is also important to consider the implications of marginal health literacy within the sociolinguistic context of metroplexes on the border such as the one in which the study was conducted. This context provides an opportunity to call into question the significance of low or marginal health literacy and Limited English Proficiency (LEP) across subpopulations of Hispanics.

The study was conducted in a U.S.-Mexico border community in which the language environment and language needs and preferences vary from other non-border communities. In the city where the study was conducted, more than 80% of the population is Hispanic/Latino, and more than 71% of the residents speak Spanish at home (U.S. Census Bureau 2014). Daily activities in all social domains are negotiated among individuals who lie along a broad continuum of Spanish and English bilingualism (Teschner 1995). What has not been sufficiently explored in the literature is whether English proficiency and functional health literacy in English, as measured using currently available instruments, are essential to obtaining reliable health information and accessing the health care system in a community in which most people have learned to navigate a bicultural and bilingual environment and are familiar with the established health care systems (on both sides of the border). Although studies have found that LEP constitutes a barrier to health care and is associated with poorer health status in Hispanics (Pippins, Alegría & Haas 2007), whether this is true among the border communities of this study and other border communities is an issue in need of further investigation. It is possible that in communities in which there is not a pressing need for learning and using English to carry out normal daily activities, and in which meaning negotiation among bilinguals is the norm, including within the available health care systems accessed in two countries, the connection between LEP, low English health literacy and poorer health outcomes is not as evident. However, it cannot be assumed that health literacy levels have no impact on health outcomes in this context, either. There is an urgent need for further research into border bilingual communities, and on how to measure health literacy within such richly layered social and linguistic contexts. In this particular border community, for example, the hospital systems lack professional interpreters and translators, and no research to date has explored how provider-patient interactions take place; how accessible information is in both languages; and how individuals with different levels of literacy and bilingualism navigate the system.

In summary, the language of the instrument used to assess health literacy level; the L1 and L2 proficiency of participants; and the geographic and sociolinguistic environment are variables that merit further consideration in health literacy research. Concerning the former, we may accept that, for the most part, the language of the healthcare system in the U.S. is English and that L1 Spanish speakers have lower English health literacy than native English speakers (Sentell & Braun 2012, Williams, Parker & Baker 1995). However, researchers have also recommended that future research consider whether differences in health literacy level among U.S. Hispanics is determined by language use alone or associated with cultural adaptations such as health beliefs and practices (Boyas 2013). We contend here that geography and sociolinguistic environment must also be integrated into research on health literacy. In addition, this research must go beyond implementation of health literacy tests to in-depth sociolinguistic and ethnographic analysis of particular geographic areas, including within hospital and clinical settings. These observations may apply globally to other border areas. Additionally, there are some other relevant global questions related to language acquisition and proficiency that urgently need to be addressed. First, there is general consensus among language acquisition researchers that L1 language proficiency directly influences second language (L2) acquisition. According to Cummins’ language interdependence principle, academic aspects of language proficiency, including literacy (reading and writing) of L1 proficiency, are transferable to L2 (Cummins 1991, Cummins 2012). This suggests that a bilingual person who has high literacy in L1 is likely to perform better on a test of L2 literacy. Future studies in health literacy should test for both L1 and L2 proficiency, and collect explicit data on educational attainment in L1 and L2 in order to explore the complex interdependence of literacies. Only through such rigorous, in-depth language research will it be possible to fully understand the significance of the results of literacy tests and the challenges faced by LEP populations in accessing the healthcare system throughout the U.S.

## Limitations

The results and contribution of the present study must be considered within its particular design and the characteristics of the participants, as well as the context in which the study was conducted. For instance, health literacy was assessed in English and participants included only immigrants whose L1 was Spanish with a low-to-intermediate level of English. However, the majority had a high school or higher degree, had lived in the U.S. for eight or more years, and more than half had taken English classes for more than 1 year. Thus, findings cannot be generalized to the general Hispanic population: demographic and contextual factors vary according to sociocultural and geographical context. We reemphasize here that a weakness in many health literacy studies in general has been a lack of in-depth description of study populations and settings. Without such description, it is difficult to tease apart contributors to low health literacy, and to interpret the impact on healthcare access and outcomes accurately.

This was a small exploratory study, and results may only apply to adults with the same characteristics as those of the study population. The study used a health literacy definition that includes only functional health literacy. The TOFHLA was not administered individually, but rather to an entire group. Although the content of the test was not altered, results may not be comparable to one-on-one administration. In addition, Spanish proficiency levels were collected through self-report of language used at home. Finally, only variables that were the focus of the original study were explored, which were limited. Including other independent and dependent variables may have yielded additional significant results.

## Conclusions

The results of this study emphasize the importance of considering both language context and language use in heath literacy research, particularly in the interpretation of the results of health literacy assessments. Contextual factors must be considered in studies looking at the impact of health literacy on health outcomes, including health access, health quality and health status. This is especially important in the U.S., considering the growth of the Spanish speaking population in many communities across the country, particularly along the U.S.-Mexico border region. Globally, language diversity is facilitated by globalization, immigration, and displacement of people. Language use is a contextual factor that may influence health care and health outcomes, and assessing L1 and L2 proficiency should become the norm in heath literacy studies with language minorities. Health literacy research must further explore the role of bilingualism in health literacy among Hispanics, and clarify the factors that must be considered when measuring health literacy in this group This study raises many questions in need of further investigation to clarify how language proficiency and sociolinguistic environment affect health literacy in language minority adults; proposes language approaches that may be more appropriate for measuring health literacy in these populations; and recommends further place-based research to determine whether the connection between language proficiency and health is generalizable to border communities.

## Figures and Tables

**Figure 1:**
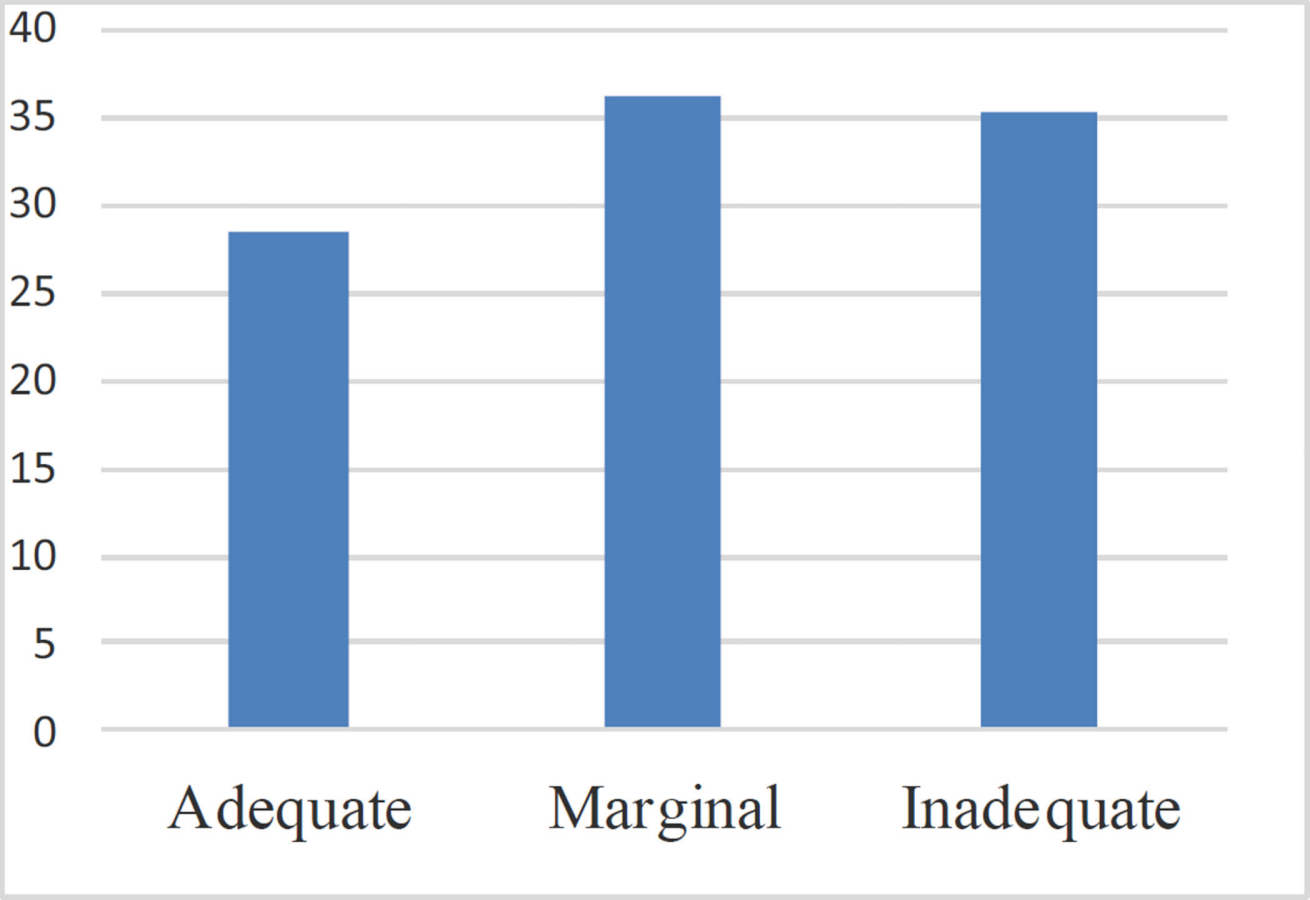
TOFHLA results, percent of respondents at each health literacy level

**Table 1:** Demographics for categorical variables (n=144)

	%	No.

Age in years		
30 or less	9.7	14
31–45	41.0	59
46–60	44.4	64
60+	4.9	7

Sex		
Male	22.2	32
Female	77.8	112

Years in the U.S.		
0–3 years	17.4	25
4–7 years	9.0	13
8 or more years	73.6	106

Education level		
Less than high school	18.8	27
High school degree	37.5	54
Post high school education	43.8	63

Language spoken at home		
Spanish	80.6	116
Both/English	19.4	28

Attended school/college in the U.S.		
Yes	38.9	56
No	61.1	88

Years of U.S. schooling		
None	61.1	88
1 or less	14.6	21
1 to 2	14.6	21
More than 2	9.7	14

Length of English classes		
1 year or less	44.4	64
More than 1 year	55.6	80

**Table 2:** Descriptive statistics for test outcomes (n=144)

	Mean	Median	SD	Min	Max
Total TOFHLA	63.7	65.0	16.5	24.0	94.0
Numeracy (wt)	29.9	30.0	10.2	3.0	48.0
Reading comprehension	33.8	35.0	9.0	0.0	50.0
English proficiency	99.8	101.0	5.6	90.0	116.0

**Table 3:** Average total TOFHLA, reading, and numeracy test scores across different demographic predictor variables.

	Total TOFHLA Mean 95% CI	Numeracy (wt) Mean 95% CI	Reading comprehension Mean 95%CI

English proficiency score			
94.4	54.68 [51.53,57.83]	26.79 [24.55,29.03]	27.89 [26.33,29.44]
100.0	63.98 [61.72,66.23]	30.02 [28.41,31.62]	33.96 [32.85,35.07]
105.6	73.28 [70.03,76.52]	33.24 [30.93,35.55]	40.03 [38.43,41.63]
	p* < 0.001	p < 0.001	p < 0.001

Age			
35.5	64.67 [60.89,68.44]	31.32 [28.99,33.64]	33.35 [31.28,35.42]
45.5	63.66 [60.94,66.39]	29.86 [28.18,31.54]	33.80 [32.31,35.30]
55.5	62.66 [58.71,66.60]	28.40 [25.97,30.83]	34.26 [32.09,36.43]
	p = 0.468	p = 0.088	p = 0.551

Sex			
Male	66.69 [60.93,72.45]	32.25 [28.68,35.82]	34.44 [31.26,37.62]
Female	62.86 [59.78,65.94]	29.26 [27.35,31.16]	33.60 [31.90,35.30]
	p = 0.248	p = 0.146	p = 0.646

Years in U.S.			
0–3 years	62.0 [55.45,68.55]	29.16 [25.09,33.23]	32.84 [29.34,36.44]
4–7 years	60.08 [51.00,69.16]	27.92 [22.28,33.56]	32.15 [27.16,37.15]
8 or more years	64.56 [61.38,67.74]	30.35 [28.37,32.32]	34.21 [32.46,35.96]
	p = 0.559	p = 0.668	p = 0.634

Education level			
Less than high school	59.86 [53.60,66.17]	28.78 [24.86,32.70]	31.11 [27.67,34.55]
High school	64.50 [60.06,68.94]	30.35 [27.58,33.12]	34.15 [31.72,36.58]
More than high school	64.67 [60.55,68.78]	30.05 [27.48,32.61]	34.62 [32.37,36.87]
	p = 0.413	p = 0.804	p = 0.228

Language spoken at home			
Spanish	63.54 [60.50,66.58]	30.01 [28.12,31.90]	33.53 [31.87,35.20]
English/Both	64.39 [58.20,70.58]	29.57 [25.73,33.41]	34.82 [31.43,38.22]
	p = 0.808	p = 0.84	p = 0.502

Attended school/college in U.S.			
Yes	65.30 [60.94,69.67]	30.75 [28.04,33.46]	34.55 [32.16,36.95]
No	62.69 [59.21,66.17]	29.40 [27.24,31.56]	33.30 [31.38,35.21]
	p = 0.357	p = 0.442	p = 0.419

Years of U.S. schooling			
None	62.69 [59.16,66.20]	29.40 [27.22,31.57]	33.30 (31.37,35.22)
1 or less	65.38 [58.21,72.55]	31.57 [27.12,36.03]	33.81 [29.87,37.75]
1 to 2	66.19 [59.02,73.36]	30.43 [25.97,34.88]	35.76 [31.33,39.70]
More than 2	63.86 [55.07,72.64]	30.0 [24.55,35.45]	33.86 [29.04,38.68]
	p = 0.799	p = 0.846	p = 0.744

Length of English classes			
1 year or less	60.80 [56.75,64.84]	29.22 [26.68,31.75]	31.58 [29.38,33.77)
More than 1 year	66.04 [62.42,69.65]	30.49 [28.22,32.76]	35.55 [33.59,37.51]
	p = 0.058	p = 0.462	p = 0.009

* p-values correspond to a test of no difference in average mean across levels of the predictor from a linear regression model. For continuous variables (age and English proficiency), the estimated mean and confidence intervals are shown when the predictor is set to the average and ±1 standard deviation from the average.

**Table 4:** Multiple regression results.*

	Total TOFHLA	Numeracy	Reading Comprehension

Estimated change in English score	9.49	3.42	6.04
95% CI for the estimated change (5.6 point increase)	7.24,11.73	1.83, 5.01	4.93, 7.15
p-value**	0.000	0.000	0.000

Estimated change in age	−2.16	−1.86	-
95% CI for the estimated change (10 year increase)	−4.37, .14	−3.46,−.26	
p-value**	0.066	0.023	

* The results correspond to changes in the average outcome for a 1 standard deviation increase in the predictor.

** p-value corresponding to the null hypothesis of no change as a function of the predictor.

**Table 5:** Row percents and 95% confidence intervals for categories of total TOFLHA score across quartiles of English language score.*

Quartiles of English score	Inadequate functional health literacy	Marginal functional health literacy	Adequate functional health literacy
1	62.5 (46.58, 76.11)	32.5 (19.77, 48.47)	5.00 (1.23, 18.16)
2	40.48 (26.71, 55.92)	40.48 (26.71, 55.92)	19.05 (9.74, 33.91)
3	19.35 (8.88, 37.16)	35.48 (20.71, 53.66)	45.16 (28.7, 62.76)
4	09.68 (3.11, 26.34)	35.48 (20.71, 53.66)	54.84 (37.24, 71.3)
Total	35.42 (27.96, 43.66)	36.11 (28.6, 44.36)	28.47 (21.63, 36.47)

* Pearson chi-square p-value for testing the null of no association between quartiles of English score and categories of TOFLHA score <0.0001.
